# NEEDS FOR SUPPORTING PEOPLE AGING WITH HIV: RESULTS FROM A CBPR COMMUNITY-NEEDS ASSESSMENT

**DOI:** 10.1093/geroni/igac059.2704

**Published:** 2022-12-20

**Authors:** Andy Rapoport, Rick Guasco, Teresa Musolino, Melanie Reese, Kenneth Teasley, Margaret Danilovich

**Affiliations:** CJE SeniorLife, Chicago, Illinois, United States; CJE SeniorLife, Chicago, Illinois, United States; CJE SeniorLife, Chicago, Illinois, United States; CJE SeniorLife, Chicago, Illinois, United States; CJE SeniorLife, Chicago, Illinois, United States; CJE SeniorLife, Chicago, Illinois, United States

## Abstract

Nearly half of people living with HIV in the U.S. are over age 50 and are navigating age-related changes while managing the complexities and challenges of HIV. People aging with HIV are an emerging community—it is imperative to understand the key issues faced by this community so to improve health outcomes and eliminate health disparities. This paper presents a CBPR approach by the SHARE board (a community advisory group comprised of older people living with HIV) and academic researchers. SHARE members conducted a mixed-methods needs assessment using a semi-structured interview guide they had developed, as well as a survey to determine community priorities for research, healthcare, and social services for people aging with HIV. SHARE members conducted 32 semi-structured interviews with individuals from across the country. From the 216 survey responses (mean age 55 years), 35% were Black, 30% were Hispanic, 50% were male, and 43% had been living with HIV for 11–15 years. 44% were fearful that long-term HIV medications will adversely affect them with aging. Triangulating interviews and surveys, financial strains and caregiving were primary age-related concerns. Affording medication and transportation were primary barriers to accessing healthcare. Most respondents had experienced stigma due to both age and HIV status. Respondents felt the most important areas for future research were optimizing management of multiple co-morbidities including HIV, and cognitive changes with HIV. Results from this community-led needs assessment identified key areas for interventions and research to address conditions disproportionately affecting the health of those aging with HIV.

